# Evidence for Co-evolutionary History of Early Diverging Lycopodiaceae Plants With Fungi

**DOI:** 10.3389/fmicb.2019.02944

**Published:** 2020-01-15

**Authors:** Gian Maria Niccolò Benucci, Delaney Burnard, Lara D. Shepherd, Gregory Bonito, Andrew B. Munkacsi

**Affiliations:** ^1^Department of Plant, Soil and Microbial Sciences, Michigan State University, East Lansing, MI, United States; ^2^School of Biological Sciences, Victoria University of Wellington, Wellington, New Zealand; ^3^Museum of New Zealand Te Papa Tongarewa, Wellington, New Zealand

**Keywords:** high-throughput amplicon sequencing, early diverging plants, 16S rDNA, 18S rDNA, Endogonales, arbuscular mycorrhizal fungi, rhizobiome, Lycopodiaceae

## Abstract

Lycopods are tracheophytes in the Kingdom Plantae and represent one of the oldest lineages of living vascular plants. Symbiotic interactions between these plants with fungi and bacteria, including fine root endophytes in Endogonales, have been hypothesized to have helped early diverging plant lineages colonize land. However, attempts to study the lycopod rhizobiome in its natural environment are still limited. In this study, we used Illumina amplicon sequencing to characterize fungal and bacterial diversity in nine Lycopodiaceae (club moss) species collected in New Zealand. This was done with generic fungal ITS rDNA primers, as well as Endogonales- and arbuscular mycorrhizal fungi (AMF)-selective primer sets targeting the 18S rDNA, and generic bacterial primers targeting the V4 region of the 16S rDNA. We found that the Lycopodiaceae rhizobiome was comprised of an unexpected high frequency of Basidiomycota and Ascomycota coincident with a low abundance of Endogonales and Glomerales. The distribution and abundance of Endogonales varied with host lycopod, and included a novel taxon as well as a single operational taxonomic unit (OTU) that was detected across all plant species. The Lycopodiaceae species with the greatest number and also most unique OTUs was *Phlegmariurus varius*, while the plant species that shared the most fungal OTUs were *Lycopodiella fastigiatum* and *Lycopodium scariosum*. The bacterial OTU distribution was generally not consistent with fungal OTU distribution. For example, community dissimilarity analysis revealed strong concordance between the evolutionary histories of host plants with the fungal community but not with the bacterial community, indicating that Lycopodiaceae have evolved specific relationships with their fungal symbionts. Notably, nearly 16% of the ITS rDNA fungal diversity detected in the Lycopodiaceae rhizobiome remained poorly classified, indicating there is much plant-associated fungal diversity left to describe in New Zealand.

## Introduction

Within root tissues of terrestrial plants exists complex communities arising from intricate interactions and associations with a diverse range of microorganisms (Shakya et al., 2013; Yang et al., 2013; Wani et al., 2015). Endophytic fungi and bacteria are essential components of these plant tissues, known as the root endosphere (Shakya et al., 2013). Some endophytic species are known to protect their host plant against disease, while others contribute to plant host growth, health and overall survival (Shakya et al., 2013; Liao et al., 2019). However, relationships between microbes in the endosphere are complex, as they are multipartite and as may have indirect effects on the host and other microbes (Yang et al., 2013; Bonito et al., 2019). Further, identification of these microorganisms and understanding their interactions and dynamics is difficult due to methodological limitations in their cultivatability or detection (Appuhn and Joergensen, 2006; Lee et al., 2008; Compant et al., 2010; Podolich et al., 2015).

The evolution of fungal symbiosis with land plants was vital to the initial expansion of plants into terrestrial environments and the later diversification of land plants (Pirozynski and Malloch, 1975; Winther and Friedman, 2008; Humphreys et al., 2010; Bidartondo et al., 2011; Horn et al., 2013). As the majority of land plants require this symbiosis to survive (Redecker et al., 2000), the importance of arbuscular mycorrhizal fungi (AMF) in the endosphere is paramount. While AMF are mostly considered to be host-generalists (Davison et al., 2015), plant specificity has been observed for some species (Helgason et al., 2002; Vandenkoornhuyse, 2002; Appoloni et al., 2008). Given the high level of endemism in New Zealand and the fact that AMF and Lycopodaceae radiated contemporaneously (Lutzoni et al., 2018), novel AMF diversity remains to be uncovered in below-ground tissues of New Zealand lycopods (Gordon, 2013).

Arbuscular mycorrhizal fungi were initially considered to be the only root associated fungi belonging to Mucoromycotina (Bonfante and Genre, 2008; Öpik et al., 2010; Van Geel et al., 2014; Spatafora et al., 2016). However, the identification of other fungal associations in Mucoromycotina with early land plant lineages and new fungal fossils has sparked great interest in the origin of plant-fungal associations (Desirò et al., 2013, 2015; Truong et al., 2017). Recent identification of Endogonales and other Mucoromycotina symbioses in vascular, non-vascular, and fossil plants suggest there is also an ancient relationship between land plants and some cryptic Mucoromycotina fungi (Field et al., 2015; Rimington et al., 2015; Desirò et al., 2017; Strullu-Derrien et al., 2018).

One family of plants that has drawn attention recently are Lycopodiaceae, an ancient family of vascular land plants called club mosses (Wikström and Kenrick, 1997, 2000, 2001; Benca, 2014). Fossil records place an approximate age of 390 million years for the split between the Lycopodiaceae and its sister families Isoetaceae and Selaginellaceae (Wikström and Kenrick, 2001). Due to their morphological conservation, Lycopodiaceae are considered a relict group (Benca, 2014), with living species closely resembling that of fossils, and their modern morphological diversity is very low (Wikström and Kenrick, 1997, 2001). Lycopodiaceae are known to have fungal associations with Glomeromycotina and Mucoromycotina taxa (Rimington et al., 2015). Interestingly, the organ colonization and morphology of these fungal associates in Lycopodiaceae was unlike those seen in other host plant species and was described as “lycopodioid mycothallus interactions” rather than mycorrhizal associations (Schmid and Oberwinkler, 1993; Read et al., 2000; Horn et al., 2013; Rimington et al., 2015). The combination of both Mucoromycotina and Glomeromycotina colonization is believed to be the reason for the unique observations seen in Lycopodiaceae fungal colonization in the past (Rimington et al., 2015).

However, non-mycorrhizal fungi (Dikarya) are also present in Lycopodiaceae, regardless of AMF colonization (Winther and Friedman, 2008; Horn et al., 2013). A combination of AMF, Dikarya and bacteria could also influence the strange colonization visualized in Lycopodiaceae roots previously. Endospheric associations of native New Zealand Lycopodiaceae are limited and have not been assessed together for fungi and bacteria (Rimington et al., 2015). Endospheric associations of bacteria are thought to be more specific than those that occur in the rhizosphere (Podolich et al., 2015). Through comparative metagenomic studies between hosts, bacterial pathogens specific to *Selaginella* have been identified (Dang et al., 2019). Five genera of Lycopodiaceae are recognized in New Zealand (Øllgaard, 1987; Burnard et al., 2016), which contain 11 species. None of these species are endemic. Given the age of this ancient family and New Zealand’s geographic isolation from other land masses, there is potential for unique and undocumented endophytic fungal and bacterial communities in Lycopodiaceae.

The aims of this paper are to assess Mucoromycota diversity, along with general fungal and bacterial endophytic diversity, within and between native New Zealand Lycopodiaceae species. Our research questions ask (1) Do both AMF and Endogonales colonize native Lycopodiaceae roots? (2) Does bacterial and fungal composition differ between Lycopodiaceae species? (3) Is there undiscovered Mucoromycota diversity present within the rhizobiome of Lycopodiaceae?

## Materials and Methods

### Sample Collection

All the New Zealand lycopod root specimens were collected in the field specifically for this study. In this large-scale survey, 200 individual plants were sampled across the North Island and South Island (Table 1 and Supplementary Figure S1). Phylogenetically, Selaginellaceae is the closest relative to Lycopodiaceae (Rydin and Wikström, 2002) and is abundant in New Zealand (Brownsey and Smith-Dodsworth, 1989), thus the adventive *Selaginella kraussiana* was selected as the outgroup. Root sections of 5–15 cm in length were collected from each species with a total sample size of 20 roots collected for each species, cleaned on the day of collection with sterile dH_2_O (Merckx et al., 2012), and subsequently stored in silica gel.

**TABLE 1:** Geographic origin of specimens used in this analysis.

**Species**	**Cepname**	**Location**	**Coordinates**	**Altitude (m)**
*Huperzia australiana*	Hupeaust	Takaka Hill	40.9320 S 172.9147 E	840
*Lycopodiella cernua*	Lycocern	Te Puru	37.0448 S 175.5388 E	60
*Lycopodiella diffusa*	Lycodiff	Charleston	41.9355 S 171.4362 E	170
*Lycopodiella lateralis*	Lycolate	Charleston	41.9355 S 171.4362 E	170
*Lycopodium deuterodensum*	Lycodeut	Te Puru	37.0321 S 175.5906 E	600
*Lycopodium fastigiatum*	Lycofast	Takaka Hill	40.9365 S 172.9075 E	800
*Lycopodium scariosum*	Lycoscar	Takaka Hill	40.9365 S 172.9075 E	800
*Lycopodium volubile*	Lycovolu	Wellington	41.2989 S 174.7210 E	710
*Phlegmariurus varius*	Phlevari	Okarito	43.2249 S 170.1571 E	840
*Selaginella kraussiana*	Selakrau	Wellington	41.2582 S 174.7722 E	140

*These same samples were also used to resolve the phylogenetic relationships of these taxa (Burnard et al., 2016). Cepname abbreviations of species were generated following the Cornell Ecology Programs (CEP) abbreviations in the *vegan* R package (Oksanen et al., 2019), and these are used in all figures.*

### DNA Extraction

Genomic DNA was extracted from homogenized, dried root samples using a previously described cetyltrimethylammonium bromide (CTAB) chloroform extraction protocol with modifications (Bonito et al., 2014). Pelleted DNA was resuspended in 40 μL of TE buffer, quantified using a NanoDrop 2000c UV-Vis spectrophotometer (Thermo Scientific), and diluted to a concentration of 5 ng/μl with PCR grade dH_2_O.

### PCR Amplification and Sequencing

Amplicons and MiSeq libraries were generated using three distinct PCR cycles as described previously (Chen et al., 2018). Briefly, target loci were first amplified with standard primer sets for 10 cycles to enrich target PCR template for each of the four primer sets: ITS1F/ITS4 to target ITS1 Fungal rDNA, 515F/806R to target Prokaryotic rDNA, Endo18S-1F/NS6 to specifically target Endogonales 18S rDNA, and NS31/AML2 to specifically target AMF 18S rDNA (Table 2). PCR reactions contained 2.5 mM of dNTPs, 0.25 pM each primer, 1X Ex Taq buffer, 1 U Ex Taq DNA polymerase (Takara Bio), 25 μg of BSA, and 20–25 ng of DNA for each targeted locus. Thermocycling conditions for each locus are described in Supplementary Table S1. PCR products were quantified after the attachment of barcodes in the third PCR, whereby each species were tagged with a different barcode. PCR products were combined at equimolar ratios into four pools (one for each locus), and purified using Agencourt AMPure XP beads (Beckman Coulter) with a ratio of 0.8 μL magnetic beads per 1 μL of PCR product. The four pools of purified amplicons were submitted to the Australian Genome Research Facility (AGRF) where the size, concentration and purity of pooled amplicons were quantified with TapeStation (Agilent). Sequencing was performed with the MiSeq (Illumina) platform where fungal and bacterial analyses were performed over two separate runs. The 16S rDNA library was sequenced using 2 × 250 bp paired-end sequencing, while the fungal amplicon library composed of three loci at equimolar ratios was sequenced using 2 × 300 bp paired-end sequencing.

**TABLE 2:** Primers used to analyze Lycopodiaceae root biodiversity with next-generation sequencing.

**Primer**	**Direction**	**Sequence (5′-3′)**	**Target**	**Specificity**	**Sources**
NS31	Forward	TTGGAGGGCAAGTCTGGTGCC	18S rDNA	Universal Eukaryote	Kohout et al., 2014
AML2	Reverse	GAACCCAAACACTTTGGTTTCC	18S rDNA	Glomeromycotina	Kohout et al., 2014
ITS1F	Forward	CTTGGTCATTTAGAGGAAGTAA	ITS rDNA	General Fungi	White et al., 1990
ITS4	Reverse	TCCTCCGCTTATTGATATGC	ITS rDNA	General Fungi	White et al., 1990
Endo18S-1F	Forward	GAGGTGAAATTCTTGGATTTATGA	18S rDNA	Endogonales	Lutzoni et al., 2004
NS6	Reverse	GCATCACAGACCTGTTATTGCCTC	18S rDNA	General Fungi	White et al., 1990
515F	Forward	GTGCCAGCMGCCGCGGTAA	16S rDNA	General Bacteria	Neher et al., 2013
806R	Reverse	GGACTACHVGGGTWTCTAAT	16S rDNA	General Bacteria	Neher et al., 2013

### Bioinformatic Analysis

Raw forward and reverse reads were quality evaluated with FastQC. Forward and reverse 16S rDNA reads were merged using PEAR (Zhang et al., 2014), while only forward reads were used in the downstream analysis for ITS, 18S, and SSU rDNA sequences. Selected reads were separated into the different datasets based upon sequencing primers with Cutadapt (Martin, 2011), quality-filtered and trimmed to equal length (Edgar and Flyvbjerg, 2015; Edgar, 2016b), de-replicated, and clustered into operational taxonomic units (OTUs) based on 97% similarity with the UPARSE (Edgar, 2013) algorithm. The taxonomic assignment for the ITS marker was performed in CONSTAX (Gdanetz et al., 2017) using the UNITE (Nilsson et al., 2019) reference database v. 07-12-17. For 16S rDNA, we used the RDP Naïve Bayesian Classifier (Wang et al., 2007) Release 2.11 against the RDP 16S and 18S rDNA training sets as well as SINTAX (Edgar, 2016a) against the SILVA (Quast et al., 2013) reference database v.123 to better assess for presence of mitochondria, plastid, and other non-target sequences. For 18S rDNA, we used SINTAX against SILVA v.123 (Clark et al., 2016), and then manually corrected assigned taxonomies by comparing each OTU sequence to those present in GenBank (Sayers et al., 2019) with BLAST (Altschul et al., 1990). OTUs were imported into R (R Core Team, 2018) and additionally filtered to discount non-target sequences, PCR errors and Illumina cross-talk errors.

### Statistical Analysis

Operational taxonomic unit distributions for the four loci and 11 plant species were compared using *phyloseq* (McMurdie and Holmes, 2013) and plotted in *ggplots* (Wickham, 2009). Cluster dendrograms were calculated starting from the plant genetic distance matrix, calculated from plant species chloroplast *rbc*L sequences (Burnard et al., 2016), and the microbial community Bray–Curtis (Bray et al., 1957) dissimilarity matrix using the *stats* package (R Core Team, 2018). Tanglegram analysis, a visual method to compare two trees with the same set of tip labels connected by lines, were plotted using the *dendextend* package (Galili, 2015) to compare plant and microbial dendrograms. The quality of the alignment of the two trees was quantified via the entanglement that varies between 1 (full entanglement) and 0 (no entanglement); a lower entanglement coefficient corresponds to a good alignment. Penalty was assessed using *L* = 1 whereby increased L results in increased penalty for sharp angles. Mantel tests implemented in *vegan* (Oksanen et al., 2019) were used to assess the correlation between matrices, where the amplicon data were normalized to the minimum library size for each dataset. Principal coordinates analysis (PCoA) was used to asses difference in community similarity in the ordination space (for the first two axis) between different plants using the Bray–Curtis distance as implemented in *phyloseq* (McMurdie and Holmes, 2013). A heatmap tree for the ITS dataset showing log2-fold change differentially abundant [ratio calculated as *log*2(*median*(*x*_1_)/*median*(*x*_2_))] taxa between all the Lycopodiaceae relative to the outgroup (*S. kraussiana*) was generated using the *metacoder* (Foster et al., 2017) R package. Upset diagrams to detect unique and shared OTUs between sets of samples (i.e., plant species) were calculated with the R package *UpSetR* (Conway et al., 2017). To assess the relationship between OTU number and DNA reads for the major microbial groups, species accumulation curves were generated using the R package *iNext* (Hsieh et al., 2019).

## Results

### Sequencing Depth and Quality

A total of 21,625,315 fungal sequences were retrieved from the first sequencing run and 19,008,928 bacterial sequences were retrieved from the second sequencing run. After the bioinformatic analysis, a total of 16,481,599 fungal sequences (including ITS, and 18S rDNA markers) and 10,942,338 bacterial sequences were analyzed cumulatively for each individual MiSeq run. Detailed information on sequence and OTU numbers and accumulation curves obtained for each primer set for the major fungal groups are reported in Supplementary Table S2 and Supplementary Figure S2.

Based on preliminary numbers of sequences retrieved for each Lycopodiaceae species, it appears that species from which a large number of fungal sequences were retrieved (>2,000,000) contained a lower number of bacterial sequences (<2,000,000) and vice versa. The only exception was *Lycopodium volubile*, which appeared to have relatively low sequence retrieval for both fungi and bacteria (<1,500,000). *S. kraussiana* had the highest number of sequences retrieved for both fungi and bacteria overall. Rarefaction curves were used as a qualitative method to estimate the species richness as a function of sequencing depth (Supplementary Figure S3), which indicated that saturation of the fungal and bacterial OTUs were achieved for the nine Lycopodiaceae species and the *S. kraussiana* outgroup.

### Lycopod Root Mycobiome

**FIGURE 1:**
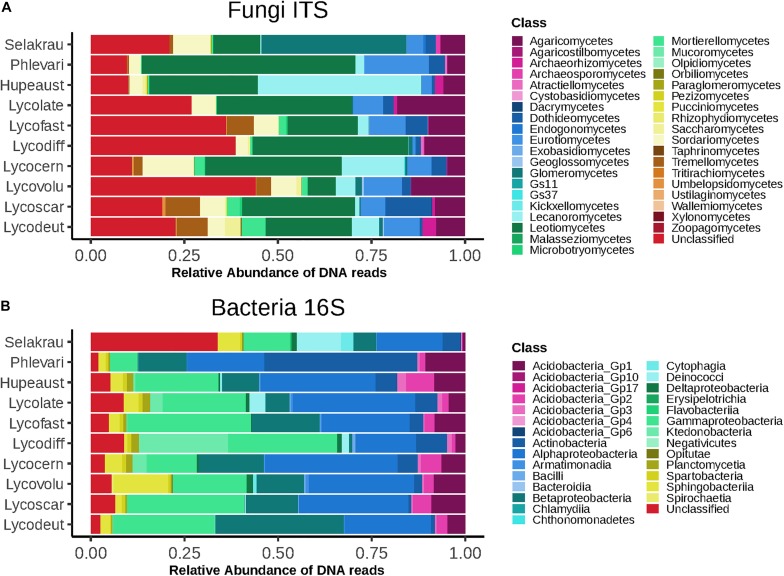
Higher level taxonomic assignments of communities at class level based on relative abundance of DNA amplicon sequences. **(A)** Fungi based on ITS sequences. **(B)** Bacteria based on 16S rDNA sequences. Samples are separated by plant host and microbial taxonomic ranks for abundant taxa are indicated on the right. GS11 and GS37 represent new classes in the Rozellomycota and Lecanoromycetes, respectively (Tedersoo et al., 2017).

The fungal community based on ITS rDNA consisted of 4616 OTUs after rarefaction. At the phylum level, Ascomycota represented 62.5%, Basidiomycota 13.9%, Rozellomycota 1.4%, and Mucoromycotina 6.7% of relative read abundance. We also recovered 15.3% fungal sequences that were unclassified at the phylum level. At class level, Leotiomycetes were most abundant (29.2%), followed by Agaricomycetes (9%) and Eurotiomycetes (7.6%). Glomeromycetes represented 4.4%, Mortierellomycetes 1.7% and Endogonomycetes 0.08% of the relative abundance (Figure 1A).

**FIGURE 2:**
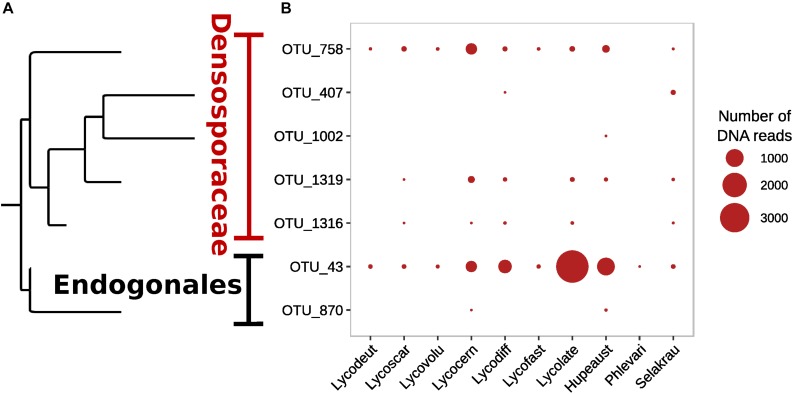
Prevalence of Endogonaceae OTUs in lycopod roots based on **(A)** phylogenetic relationships and **(B)** relative abundance of DNA of 18S rDNA amplicon sequences from the Endo18S-1F/NS6 primer pair designed to target Endogonales.

Although Glomeromycotina were targeted with the chosen 18S NS31-AML2 primers, only 24 (9.7%) out of 248 OTUs from this primer set were classified as Glomeromycotina (Supplementary Figure S4). The remaining sequences from this primer set (after removing host plant rDNA) were classified as Ascomycota (65.4%), Basidiomycota (16.9%), Chytridiomycota (2.0%), Mortierellomycotina (2.0%) and 4.0% unclassified Fungi (*incertae sedis*). Similarly, only 7 out of 408 OTUs targeted with Endo18S-1F/NS6 “Endogonales-specific” 18S rDNA primers were classified as Endogonales, including *Endogone* (OTU 43), *Jimgerdemannia* (OTU 870), *Densospora* (OTU 758), and *Sphaerocreas*-related (OTUs 407, 1002, 1316, 1319) (Figure 2). While OTU 43 was the most abundant and also identified in all lycopod species, OTU 758 was the second most abundant and it was recovered in all but one plant host. Of the seven Endogonales OTUs, three were in low abundance in only 1–2 host plant species. For comparison, five of the seven Endogonales OTUs were detected in the *S. krausianna* outgroup.

**FIGURE 3:**
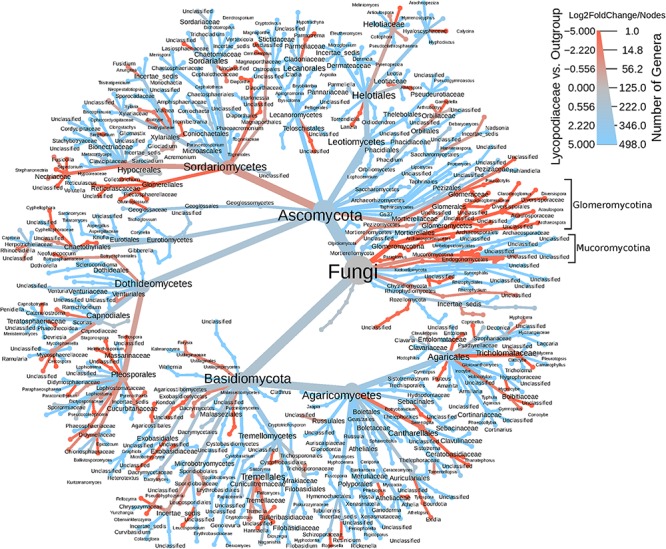
Heatmap tree of fungal genera detected in the ITS dataset for Lycopodiaceae relative to the outgroup (*Selaginella kraussiana*) and also representative of number of genera. The heatmap scale represents specificity (log2FoldChange) of taxa for Lycopodiaceae (blue) or for *S. kraussiana* (red) and indicates number of genera (nodes) underlying this specificity. Glomeromycotina and Mucoromycotina are highlighted in brackets.

In the ITS rDNA dataset, 432 OTUs were classified as Ascomycota (61.3%), Basidiomycota (15%), Chytridiomycota (6.1%), and ∼16% that were classified as *incertae sedis*. Interesting, both Endogonales and AMF were amplified and sequenced with the ITS primer set. The heatmap tree (Figure 3), based on ITS1 data, shows that Glomeromycotina, as well as Mucoromycotina (Endogonales), were enriched in *S. kraussiana* compared to the other Lycopodiaceae considered in this study. In contrast, in the 18S rDNA dataset *S. kraussiana* does not appear to be enriched in Endogonales compared to lycopods (Figure 3).

The prokaryotic community was composed of 551 OTUs after rarefaction. *Proteobacteria* dominated (61.4%), followed by Acidobacteria (10.3%), Actinobacteria (8%), Bacteroidetes (4.9%), and Chloroflexi (4%). A total of 6.8% Prokaryotes were unclassified at phylum level. At class level, Alpha*proteobacteria* dominated (25.8%), followed by Gamma*proteobacteria* (22.8%), Beta*proteobacteria* (13.2%), and Actinobacteria (7.9%) (Figure 1B).

Principal Coordinates Analysis (PCoA) ordination graphs showed differences in community structure of fungal and bacterial communities in the Lycopodiaceae species (Supplementary Figure S5). The fungal and bacterial communities of *Lycopodiella diffusa* and *Lycopodiella lateralis* clustered together and were distinct in the ordination space compared to the other plant species. Additionally, the fungal and bacterial communities of *S. kraussiana* were separate from those of other plant species.

The number of common and unique fungal OTUs was investigated with respect to plant host species (Supplementary Figure S6). There were between 656-799 fungal OTUs in most lycopod species, while fewer OTUs were in *L. lateralis* and *L. diffusa* that had 531 and 422 fungal OTUs, respectively. *Selaginella kraussiana* had the highest number of unique OTUs (727), followed by *Phlegmariurus varius* (404), and *Lycopodiella cernua* (367). The lowest number of unique species was found in *L. diffusa* (121). Species that shared the highest number of OTUs were *Lycopodiella fastigiatum* and *Lycopodium scariosum* (87 OTUs shared), *L. lateralis* and *L. diffusa* (80 OTUs shared), and *L. volubile* and *S. kraussiana* (64 OTUs shared), while intersections between other plant species shared less than 30 OTUs. In comparison, *S. kraussiana* outgroup had more fungal OTUs (1230 OTUs), although these OTUs were not specialists, as they were also found in all other lycopod species examined except *L. diffusa*. Likewise, the number of common and unique bacterial OTUs was investigated with respect to plant host (Supplementary Figure S7), although the patterns were not consistent with those of fungal OTUs. The most OTUs were in *S. kraussiana* (440 OTUs), of which 105 OTUs were unique to this plant species. All other species had relatively similar numbers of OTUs (between 264-352 OTUs) in which there were only few OTUs unique to each species. In contrast to the fungal OTUs, bacterial OTUs were less shared. Species that shared the highest number of bacterial OTUs were *L. scariosum* and *P. varius* (29 OTUs shared), followed by intersections of other species that shared less than four OTUs.

### Correlation Between Microbial Community Diversity Host Plant Genetic Distance

**FIGURE 4:**
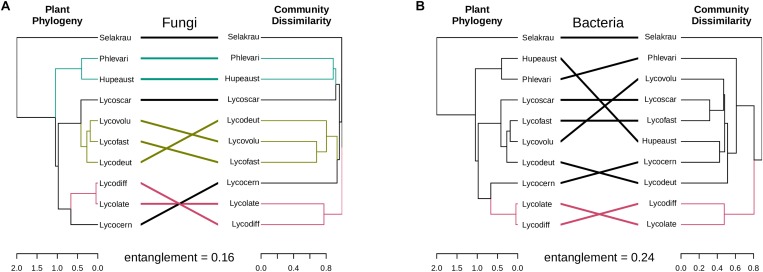
Tanglegrams showing concordance between microbial community and host plant phylogeny. Plant phylogeny based on genetic distances was constructed using MrBayes (Huelsenbeck and Ronquist, 2001) and the same plant samples collected for this study (Burnard et al., 2016). **(A)** Fungal dendrogram based on community similarities (Bray–Curtis distance) derived from ITS sequences. **(B)** Bacterial dendrogram based on community similarities (Bray–Curtis distance) derived from 16S rDNA sequences. The colored lines in the tanglegram represent “clades” that share similarity, while black lines represent random distribution between host-microbial distances.

The genetic distances between the plant species showed significant positive correlation with the fungal community dissimilarities (Mantel *r* = 0.84, *p* = 0.001, perm. = 999) based on ITS. However, no significant negative correlation between plant phylogenetic distances and microbial community dissimilarities was detected in prokaryotic rhizobiome communities (Mantel *r* = −0.42, *p* = 0.99, perm. = 999). Correlations between dendrograms based on plant genetic distance and microbial community similarities for both the fungal and prokaryotic distances are graphically visible in the tanglegram presented in Figure 4. The entanglement between the plant phylogeny and the fungal community dissimilarities was 0.16, while it was 0.24 for the prokaryotic communities. In many cases, variation in fungal community dissimilarity between *Selaginella* and lycopods (e.g., *S. kraussiana* and *L. volubile*) was lower than that between two lycopod species (e.g., *L. diffusa* and *L. volubile*). A similar trend was also detected in the bacterial dataset. For example, *S. kraussiana* communities were more similar to those of *L. lateritium* compared to those of *L. volubile* to *L. deuterodensum*. Notably in both fungal and prokaryotic tanglegrams, *L. lateralis* and *L. diffusa* clustered together in one clade in the community dissimilarity, which was consistent with the PCoA ordinations as well as the plant phylogeny.

## Discussion

There has been much interest in the mycobiome of lycopods given recent evidence that these early diverging plants host fine-root endophytes belonging to Endogonales and coarse-root endophytes belonging to Glomeromycotina, and that they may represent relic plant-fungal symbioses (Bidartondo et al., 2011; Field et al., 2016; Hoysted et al., 2019). Here we conducted the first study of Lycopodiaceae rhizosphere in New Zealand. We identified and annotated the fungal and bacterial communities of 20 representatives of each of 10 plant species. After sample rarefaction, next-generation sequencing resolved 4616 fungal OTUs and 551 bacterial OTUs. Previous studies have reported on fungi from a few lycopod species through targeted sequencing from a single plant sample. We found extensive biodiversity of both fungi and bacteria was detected in the rhizobiome of Lycopodiaceae taxa. Interestingly, fungal community similarities clustered by plant host clade, potentially reflecting shared co-evolutionary history of Lycopodiaceae plants with fungi. However, bacterial community diversity was not correlated with host genetic distance. The bacterial microbiome in our Lycopodiaceae root sections was particularly rich in alpha-, beta- and gamma-*Proteobacteria*. Nearly 16% of the fungal diversity detected in this study was poorly classified. This is partly expected due to the limitations of current databases, and limited prior sampling in Australasia and specifically the New Zealand habitats that we sampled.

Arbuscular mycorrhizal symbioses with Glomeromycotina fungi are known to be an ancient and widespread symbiosis with plants. In this study, 18S rDNA of Glomeromycotina fungi in lycopod rhizobiome samples were targeted with the NS31/AML2 primer set, which has been used previously for high-throughput amplicon sequencing studies of AMF (Kohout et al., 2014; Davison et al., 2015). To our surprise, only a small percentage (9.7%) of AMF sequences were recovered from among the millions of sequences. These sequences clustered into 24 OTUs that matched with “virtual taxa” recovered in independent studies and all but 1 OTU classified within a single family, Glomeraceae. Although the NS31/AML2 primer set was not used on Lycopodiaceae species in other studies of New Zealand lycopods, this primer set has been used extensively worldwide in other high-throughput sequencing metagenomic studies focused on AMF (Öpik et al., 2013). For example, the evaluation of NS31/AML2 on six non-Lycopodiaceae plant species obtained a total of 17 Glomeromycotina OTUs (Kohout et al., 2014). It has been noted that the NS31/AML2 primer set has a tendency to amplify large amounts of Ascomycota and Basidiomycota, which contributed to the large, and possibly overrepresented, amount of these fungi, a result that is reflected in our lycopod study as well as an independent study of liverworts (Rimington et al., 2018). The Illumina adaptors and barcodes added to the PCR primers in our study may also have interfered with their specificity, thus future research with these primers may further optimize PCR conditions. Alternatively, AMF may account for a small proportion of fungi in the lycopod rhizobiome.

Recent studies have implicated Mucoromycotina fine root endophytes in Endogonales as a second AMF lineage of ancestral fungal symbionts of land plants (Bidartondo et al., 2011; Rimington et al., 2015). Independent calibrated molecular dating studies that include Endogonales, AMF, and other early diverging lineages indicate that Endogonales originate in the mid–late Silurian (∼420 Ma), contemporaneously with AMF and lycopods (Lutzoni et al., 2018; Chang et al., 2019). Phylogenetic systematics of Endogonales have resolved two family level clades: Endogonaceae and Densosporaceae (Desirò et al., 2017). Endogonaceae consists of *Endogone* and *Jimgerdemannia*, two deeply divergent monophyletic lineages of macro sporocarp producing species, that differ in spore morphology, sporing habit, and (putatively) ecology. Interestingly, most of the molecular diversity that has been detected in Endogonales is from plant roots, rather than from sporocarpic fruiting structures. This is particularly true of fungal diversity within Densosporaceae. For example, only four species of *Densospora* and four species of *Sphaerocreas* have been described based on formal descriptions of fruiting structure, yet more than 40 taxa are estimated based on molecular phylogenetics of environmental data (Desirò et al., 2017).

While ITS is known to generally discriminate Endogonales due to amplification and sequencing challenges (Desirò et al., 2017), a subset of ITS sequences in this study of Lycopodiaceae had relatively high affinity to Endogonales including sequences with 97–100% identity to “*Mucoromycotina* sp.” sequenced from the thallus of *Treubia lacunosa* and phylogenetically classified with *Endogone* species (Bidartondo et al., 2011) as well as sequences with 90% identity to *Endogone pisiformis*. To enhance our study, Endogonales diversity was assessed by selectively amplifying and sequencing 18S rDNA using the Endo18S-1F/NS6 primer pair. Within sampled roots of lycopods and *Selaginella*, we recovered sequences that were classified as *Endogone*, *Jimgerdemannia, Densospora*, and *Sphaerocreas*. These OTUs were not dominant in any of the samples, but they were often present across multiple species. While the sequence of only one single taxon of *Endogone* matched 100% to reference sequences in NCBI, the remaining novel sequences were between 1–5 bp (<3%) dissimilar to sequences previously recovered from lycopods in New Zealand and South Africa, and fern gametophytes in Japan (Desirò et al., 2013; Rimington et al., 2015; Ogura-Tsujita et al., 2019). As this represents the minority of the investigated lycopod species, it is plausible that the Endogonales OTUs also infected plants adjacent to the collected Lycopodiaceae (Hoysted et al., 2019).

Together, these results show that most species of Lycopodiaceae and *Selaginella* plant roots assessed were co-colonized by both AMF and Endogonales. While we were not able to quantify the colonization of these fungi in the plant roots, we estimate that they are low in biomass given the low number of sequences recovered, despite multiple selective approaches. Undoubtedly, new primer sets with increased efficiency in amplifying AMF and Endogonales, combined with improved long-read sequencing technology, will enable more sensitive analyses on the diversity and distribution of AMF and Endogonales.

Assessments of the whole fungal community in Lycopodiaceae species were made with fungal-specific ITS primers and revealed that lycopods were heavily colonized by Ascomycota. Compared to the *Selaginella* outgroup, lycopods were particularly enriched in Helotiales, Coniochaetales, Sordariales and other early diverging Ascomycota. Contrary to our expectations, our ITS dataset found that *Selaginella* was enriched in AMF, Hypocreales, and Pleosporales compared to sampled Lycopodiaceae. 18S rDNA data indicate that at least four of the major clades of Endogonales are present in Lycopodiaceae and *Selaginella*. Our recovery of Pezizomycotina in this study is consistent with a previous study of Lycopodiaceae in Germany (Horn et al., 2013). Many orders of Ascomycota (e.g., Xylariales, Chaetothyriales, Helotiales), including dark septate endophytes that colonize root tissues intracellularly and intercellularly were also detected. This is consistent with reports of dark septate endophytes in belowground tissues of *Lycopodiella inundata* (Jumpponen, 2001; Fuchs and Haselwandter, 2004). We found it particularly interesting that lycopods are co-colonized by dark septate endophytic fungi, AMF and Endogonales (along with other fungi). This raises questions regarding each taxon’s respective function and reinforces the biocomplexity of mutualisms in belowground systems, which includes functional complementarity between Glomeromycotina and Mucoromycotina in ancient plant-fungal mutualisms (Field et al., 2019).

Our results also demonstrate that Ascomycota and Basidiomycota are present in Lycopodiaceae roots, regardless of AMF colonization (Winther and Friedman, 2008; Horn et al., 2013). Nor did the presence of AMF in the lycopod rhizobiome correlate with either the composition or abundance of Ascomycota and Basidiomycota. Many Ascomycota and Basidiomycota taxa have been sequenced and cultured from belowground tissues of non-vascular plants including mosses and ferns (Chen et al., 2018; Ogura-Tsujita et al., 2019). Some of these fungal taxa are thought to be multi-trophic, and may switch between biotrophic and saprotrophic modes of growth during their life cycle.

Intriguingly, Lycopodiaceae species that showed a higher abundance of Ascomycota OTUs and sequence number exhibited an inverse relationship for Basidiomycota. Parasitism or competition (e.g., niche exclusion) between these fungal groups in the rhizobiome may explain this pattern. Sebacinales are another basal order of basidiomycetes that are known to form mycorrhizal-like associations with host plants, including *Lycopodium* gametophytes in New Zealand (Horn et al., 2013). Surprisingly, we did not detect Sebacinales in the lycopod sporophytes we sampled in New Zealand. However, Tremellomycetes were the predominant class of Basidiomycota identified from lycopod roots in our study. Tremellomycetes are well known for their tendency to form mycoparasitic relationships (Bandoni, 1984). These results provide additional support for the hypothesis that Lycopodiaceae gametophytes and sporophytes have different fungal associations during their life cycle (Leake et al., 2008).

In this study, we tested whether lycopod hosts are constrained phylogenetically in the taxa or bacteria or fungi that they can associate with. We were particularly interested in early diverging AMF and fine root endophytes in the Endogonales. If plant hosts were phylogenetically constrained, we expected host phylogeny to correlate with fungal or bacterial community diversity in the rhizobiome. We found strong plant distance correlations with distance between closely related fungal taxa; however, this pattern dissolved at higher phylogenetics levels (i.e., genus/family) of the host. In contrast, no association was found between plant host genetic distance and bacterial diversity. This implies that Lycopodiaceae have evolved specific relationships with their fungal symbionts. The outgroup *S. kraussiana* further illustrated the potential for co-evolution of early land plants with diverse multi-trophic fungal species. The reciprocal impact of bacteria and fungal interactions on the evolution and distribution of taxa in the lycopod rhizobiome remains enigmatic and the subject for future research, which will consider variables such as plant compartment and biogeography (Bonito et al., 2014; Coleman-Derr et al., 2016).

Our results should be interpreted alongside the limitations of this study. First, we used Illumina MiSeq technology, which is limited in sequence read length (currently 300 bp, minus any trimming). These sequence length constraints limit taxonomic, phylogenetic and ecological inferences. Best practices may recommend sequencing the complete rDNA operon, but this approach is not yet economically feasible with current NGS technology. New long-read sequencing technologies offer promise for new opportunities in rhizobiome community analysis, but methods still need to be developed and optimized for different sample types and preparation (Ma et al., 2017). Second, DNA samples of each species were pooled to build our libraries for NGS. Thus, the lack of replicate samples limited our ability to assess intra-specific variation in rhizobiome microbial communities, including individual plants that may act more as specialists or generalists with particular fungi depending on the chemical biology of the soil and neighboring plant species. Likewise, colonization of each individual plant was not assessed by microscopy, albeit this would not have resulted in identification of microbial taxa and communities. Third, our sequencing libraries were compromised by a large amount of host and non-specific amplification and sequencing. Strategies to minimize sequence read loss due to host DNA and non-specific amplification are a constant concern in plant microbiome studies. For example, PCR blockers such as PNA clamps can be designed to limit host amplification (Jackrel et al., 2017). As sequencing technologies advance, costs decline, and PCR-free approaches emerge, our understanding on the origin and functions of plant rhizobiomes and evolutionary symbioses between plants and fungi will continue to improve.

## Conclusion

This study used targeted high-throughput amplicon sequencing to uncover extensive diversity in the root-associated microbial community of Lycopodiaceae in New Zealand. We show that Endogonales, AMF and dark septate fungi are a feature of the Lycopodiaceae rhizobiome, and that these fungi likely co-colonize and partition belowground plant tissues of other non-vascular and vascular plants. Closely related plant hosts were found to have more similar fungal rhizobiomes to each other compared to more distantly related hosts, but this was not observed for bacterial communities. Although some exact sequence variants were found for fungi in Mucoromycotina, genome resources and sequencing technologies that improve read length, depth, and quality will be required to better resolve questions of diversity, functional and evolutionary relationships between early diverging lineages of fungi and plants.

## Data Availability Statement

Raw read datasets generated for this study have been deposited in NCBI under the BioProject number PRJNA553491. The R code used in analysis of the data is available in GitHub at https://github.com/Gian77/Scientific-Papers-R-Code/tree/master/Benucci_etal_2019_Lycopods.

## Author Contributions

DB, LS, GB, and AM conceived and designed the project. DB and LS collected plant samples in the field. DB, LS, and AM conducted the laboratory methods required for next-generation sequencing. GMNB and GB completed the bioinformatic and statistical analyses of next-generation sequencing data. GMNB, DB, GB, and AM wrote the manuscript. All authors edited and approved the manuscript for submission.

## Conflict of Interest

The authors declare that the research was conducted in the absence of any commercial or financial relationships that could be construed as a potential conflict of interest.
