# Supplementary material for: Evidence for Co-evolutionary History of Early Diverging Lycopodiaceae Plants With Fungi
Source: Front Microbiol. 2020 Jan 15;10:2944. doi: 10.3389/fmicb.2019.02944 (PMC6974469; doi:10.3389/fmicb.2019.02944)
Supplement: Supplementary file 1 [file Data_Sheet_1.docx]

Supplementary Material

# Supplementary Figures

**Figure S1.** Geographic distribution of Lycopodiaceae root specimen collection sites in the North Island and South Island in New Zealand.


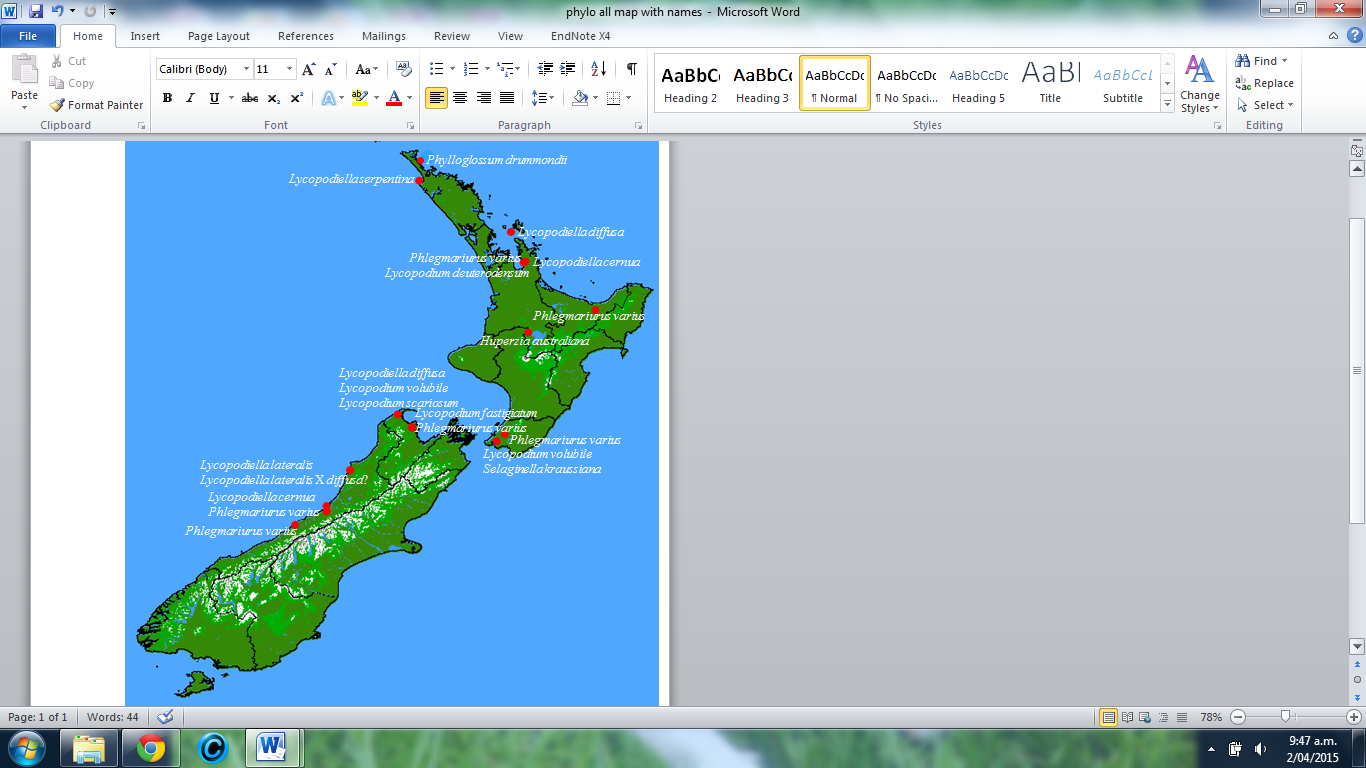


**Figure S2.** Accumulation curves for each one of the different primer sets used in this study. Single curves for Glomeromycotina, Ascomycota, Basidiomycota, and Endogonales were provided for ITS1F/ITS4, Endo18S-1F/NS6, and NS31/AML2. For the ITS data, a rarefaction curve for ‘All Fungi’ in the Kingdom is provided. For 515F/806R, single curves for All Bacteria, plastids, and mitochondria were included.

**Figure S3.** Comparison of OTU richness and sequencing coverage for host plants using rarefaction curves.

**Figure S4.** 18S rDNA phylogenetic relationships of Glomeraceae OTUs identified in this study compared to reference ‘Virtual OTUs’ of arbuscular mycorrhizal fungi.


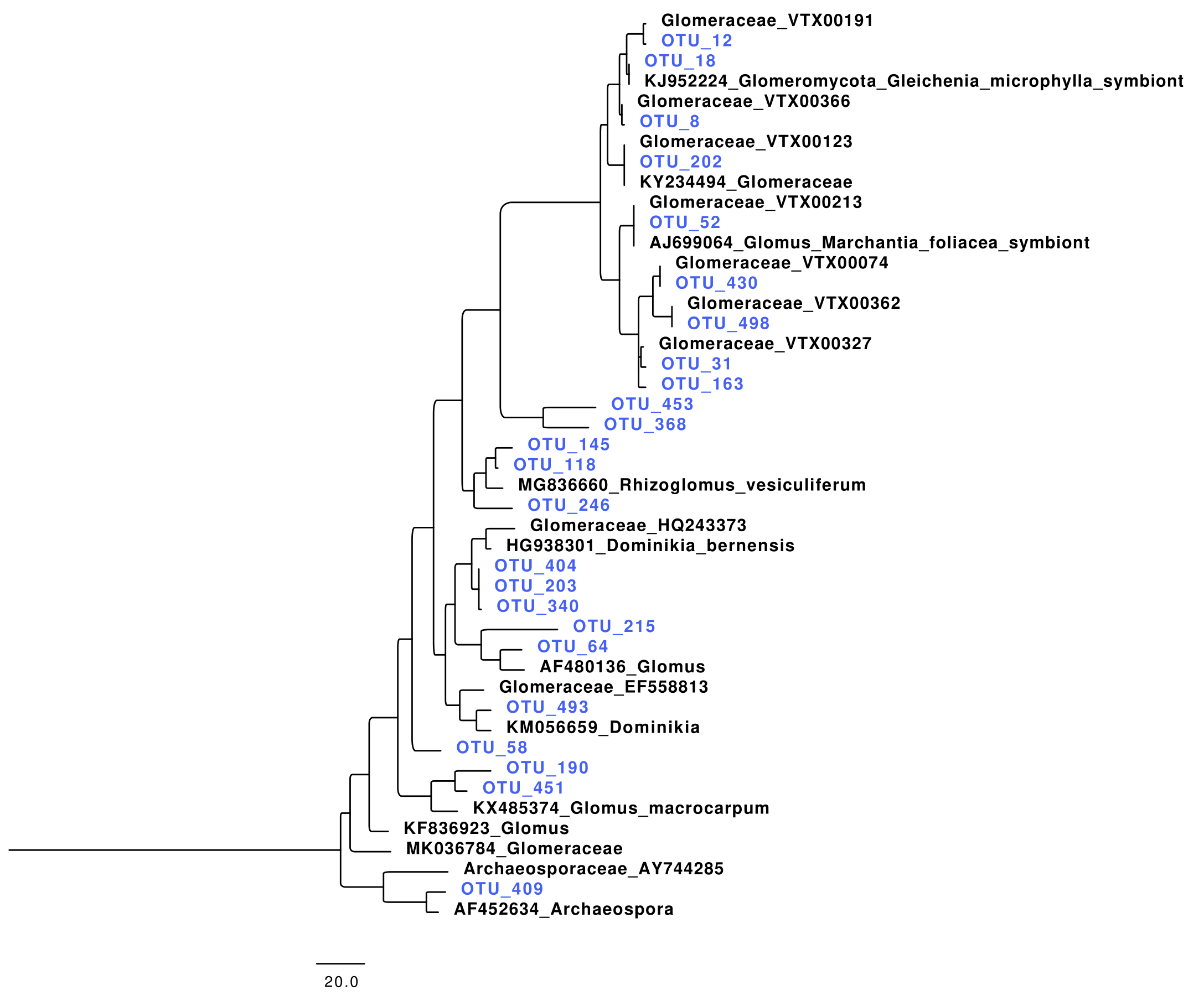


**Figure S5.** Principal Coordinates Analysis (PCoA) ordination graphs, using Bray-Curtis distance, showing differences in community structure of fungal and bacterial communities.

**Figure S6.** Similarities and differences in fungal OTUs in the Lycopodiaceae rhizobiome. Total number of OTUs are indicated for each plant species (lower left), while the vertical histogram illustrates number of OTUs unique to individual plant species or shared by two plant species. Plant species are shown in the dot matrix, where each circle point represents a plant species and lines between two points indicates the two species that share the OTUs. For simplicity, only intersections between two species are reported in the graphs.

**Figure S7.** Similarities and differences in bacterial OTUs in the Lycopodiaceae rhizobiome. Total number of OTUs are indicated for each plant species (lower left), while the vertical histogram illustrates number of OTUs unique to individual plant species or shared by two plant species. Plant species are shown in the dot matrix, where each circle point represents a plant species and lines between two points indicates the two species that share the OTUs. For simplicity, only intersections between two species are reported in the graphs.

# Supplementary Tables

**Table S1.** PCR conditions for each primer set used in PCR reaction number one in preparation for next-generation sequencing.

| **rDNA Locus** | **Primer Set** | **Reaction Volume (μL)** | **Template DNA (ng)** | **Thermocycling Parameters** | **Thermocycling Reference** |
| --- | --- | --- | --- | --- | --- |
| 16S | 515F/806R | 12.5 | 20 | 3 m at 94°C, 10 cycles of: 30 s at 94°C, 30 s at 50°C, 40 s at 72°C, followed by 10m at 72°C | Modified from Flores et al., 2012 |
| ITS | ITS1F/ITS4 | 12.5 | 20 | 10 m at 94°C, 15 cycles of: 30 s at 94°C, 30 s at 52°C, 30 s at 72°C, followed by 5 m at 72°C | Modified from Munkacsi et al., 2007 |
| 18S | NS31/AML2 | 25 | 25 | 3 m at 94°C, 20 cycles of: 30 s at 94°C, 30 s at 52°C, 45 s at 72°C, followed by 10m at 72°C | Modified from Kohout et al., 2014 |
| 18S | Endo18S-1F/NS6 | 12.5 | 20 | 5 m at 94°C, 10 cycles of: 30 s at 94°C, 30 s at 58°C, 1 m at 72°C, followed by 10m at 72°C | Modified from Desirò et al., 2013 |

**Table S2.** Summary of the reads obtained for each fungal and bacterial target group by primer combination. The number of OTUs is indicated in parentheses.

|  | **Fungi** | | | |
| --- | --- | --- | --- | --- |
|  | **All reads** | **ITS1F/ITS4** | **Endo18S-1F/NS6** | **NS31/AML2** |
| **Raw reads** | 21625315 | 16481599 | 4362555 | 359945 |
| **in OTU table** |  | 8078430 (5679)* | 186875 (432) | 86472 (248) |
| **Glomeromycotina** |  | 454175 (293) | 232 (9) | 790 (9) |
| **Ascomycota** |  | 5071752 (2855) | 56459 (51) | 976 (7) |
| **Basidiomycota** |  | 1117384 (1032) | 20808 (26) | 418 (4) |
| **Endogonales** |  | 5965 (11) | 5933 (7) | 0 |
| **Others** |  | 1429154 (1549) | 103443 (338) | 84288 (228) |
|  |  |  |  |  |
|  | **Bacteria** | | | |
|  | **All reads** | **515F-803R** |  |  |
| **Raw reads** | 19008928 |  |  |  |
| **in OTU table** |  | 10942338 (609) |  |  |
| **Bacteria** |  | 1861028 (579) |  |  |
| **Plastids** |  | 6097819 (19) |  |  |
| **Mitochondria** |  | 2983491 (11) |  |  |
